# The extraordinary satellitome diversity of freshwater crayfish: a driver of genome evolution

**DOI:** 10.1186/s13100-026-00399-8

**Published:** 2026-04-28

**Authors:** Lena Bonassin, Ljudevit Luka Boštjančić, Christelle Rutz, Caterina Francesconi, Leonie Schardt, Damian Baranski, Carola Greve, Lucian Pârvulescu, Višnja Besendorfer, Jelena Mlinarec, Ivana Maguire, Kathrin Theissinger, Odile Lecompte

**Affiliations:** 1 Department of Computer Science, ICUBE, UMR 7357, University of Strasbourg, CNRS, Centre de Recherche en Biomédecine de Strasbourg, Rue Eugène Boeckel 1, Strasbourg, 67000 France; 2 LOEWE Centre for Translational Biodiversity Genomics (LOEWE-TBG), Senckenberganlage 25, Frankfurt, 60325 Germany; 3 Ecology Department, Faculty of Biology, Rhineland-Palatinate University of Technology (RPTU) Kaiserslautern-Landau, Erwin-Schrödinger-Str. 14, Kaiserslautern, 67663 Germany; 4 Department of Experimental Medical Science, Lund Stem Cell Center, Lund University, Sölvegatan 19, Lund, 221-84 Sweden; 5 Senckenberg Research Institute and Natural History Museum Frankfurt/M, Senckenberganlage 25, Frankfurt am Main, 60325 Germany; 6 Department of Biology, Faculty of Chemistry, Biology, Geography, West University of Timisoara, Str. Pestalozzi 16A, Timisoara, 300115 Romania; 7 Institute for Advanced Environmental Research,Crayfish Research Centre, West University of Timisoara, Oituz 4, Timisoara, 300086 Romania; 8 Department of Biology, Faculty of Science, University of Zagreb, Horvatovac 102a, Zagreb, 10000 Croatia; 9 Oikon Ltd.—Institute of Applied Ecology, Trg senjskih uskoka 1-2, Zagreb, 10000 Croatia; 10 Kassel Institute for Sustainability, Institute for Biology, University of Kassel, Mosenthalstrasse 8, Kassel, 34117 Germany

**Keywords:** Satellite DNA, Repetitive elements, Decapoda, Library hypothesis, Concerted evolution

## Abstract

**Background:**

Repetitive elements, particularly satellite DNA (satDNA), play a significant role in genome evolution and organisation. However, their diversity and evolutionary dynamics remain poorly understood in non-model organisms. Freshwater crayfish (Decapoda, Astacidea) have large genomes with a high chromosome number and are rich in satDNAs. This makes them attractive for studying the impact of satDNA on genome evolution.

**Results:**

In this study, we investigated the repetitive genomic landscape of 19 species representing four freshwater crayfish families. Our analysis revealed a high proportion of repetitive DNA in all studied species, with the total repeat content ranging from 30% to 66%. The number of satDNA families was remarkably high (54–622 families per species), with minisatellites (< 100 bp) forming the largest component of the satellitome. Family-specific patterns emerged: Astacidae and Cambaroididae showed the highest satDNA proportions, while Cambaridae and Parastacidae were dominated by Class I transposable elements. Species of the family Parastacidae showed the largest number of unique satDNA clusters and were clearly separated from other families, reflecting their phylogenetic divergence and distinct biogeographic history. We identified specific satDNAs conserved across all species, among them the PlSAT3-411, pointing to their important functional roles as pericentromeric satDNA.

**Conclusion:**

This study provides the first comprehensive comparative analysis of satDNA in freshwater crayfish. Our results highlight the dynamic nature of repetitive DNA and underscore its importance in genome organisation and evolutionary history.

**Supplementary Information:**

The online version contains supplementary material available at 10.1186/s13100-026-00399-8.

## Background

Freshwater crayfish are a group of taxonomically diverse decapod crustaceans belonging to two superfamilies: Astacoidea (families Astacidae, Cambaridae, Cambaroididae) and Parastacoidea (family Parastacidae) [1]. Their distribution range spans all continents except Antarctica and Africa (with the exception of Madagascar), with particularly high diversity in southeastern North America and southeastern Australia [2, 3]. Based on phylogenetic evidence, the two superfamilies Astacoidea (in the Northern hemisphere) and Parastacoidea (in the Southern hemisphere) split around 261/268 Mya – 241 Mya [4, 5]. Within the superfamily Astacoidea, the oldest family Cambaroididae is distributed in East Asia, while the family Astacidae is distributed in Europe, with the exception of the genus *Pacifastacus*, which is found in western North America. The family Cambaridae, considered the youngest freshwater crayfish family, is distributed in eastern North America [2]. Freshwater crayfish play crucial roles in aquatic ecosystems worldwide. They are considered keystone species and ecosystem engineers, contributing to various ecological processes in their environment [6]. In recent decades, population numbers of many freshwater crayfish species have experienced severe declines due to diverse anthropogenic influence [7, 8] and are the subject of many conservation efforts [9–12].

Freshwater crayfish families exhibit high diversity in their genomic characteristics [13, 14]. Chromosome numbers in the infraorder Astacidea range from 2n = 102 in *Procambarus digueti* (Cambaridae) up to 2n = 276 in *Procambarus virginalis* (Cambaridae) [15]. This chromosomal diversity is further highlighted by significant variations in genome size and repetitive DNA content across different species and habitats. Within the infraorder Astacidea, freshwater crayfish generally possess larger genomes than the species occurring in shallow and deep water marine environments [16]. In particular, genome sizes in freshwater crayfish range from 3.16 Gb in *Cherax quadricarinatus* (Parastacidae) to 19.2 Gb in *Astacus astacus* (Astacidae), while in marine species, genome sizes range between 4.16 Gb in *Hommarus gammarus* and 4.79 Gb in *Nephrops norvegicus* [16]. A large portion of these giant genomes consists of repetitive elements (REs), with satellite DNA (satDNA) families being particularly abundant in freshwater crayfish [13, 15]. Thus, freshwater crayfish represent an interesting model for investigating the processes that drive the evolution of repeat-rich genomes.

The repetitive fraction of any eukaryote genome plays a crucial role in genome organisation, functioning and evolution [17]. Repetitive DNA can broadly be divided into transposable elements (TEs) and satDNA [18]. TEs, including retrotransposons and DNA transposons, are characterised by their ability to proliferate, and are therefore widely dispersed within the genome [19]. They can play a role in genome reorganisation, in regulation of gene expression and epigenetic processes [19]. SatDNA plays a crucial role in the formation of chromosomal structures and in genome stability, forming long tandem arrays primarily in telomeric, centromeric and pericentromeric regions of the genome [20]. In most animal and plant species, satDNA contributes to the formation of centromeres [21]. These long arrays allow the attachment of histones essential in the formation of the kinetochore [21]. The ability of satDNAs to undergo homogenisation and rapid evolution is essential for maintaining their structural and functional roles in centromeric regions [22]. Pericentromeric satDNA PlSAT3-411 has been previously identified and characterised in the crayfish *Pontastacus leptodactylus*, where it has been found to colocalise with the pericentromeric regions on all chromosomes [15]. In the same species, the PlSAT57-664 satDNA showed complex units that include the complete PlSAT3-411 unit and four direct sub-repeats, but with lower abundance than the PlSAT3-411 [15]. The most abundant satDNAs are often implicated in the crucial and conserved function of centromeres. Despite their importance, centromeric sequences often do not share conserved properties such as monomer length, GC content, or common sequence motifs [23]. These findings underscore the significance of satDNA organisation in understanding the evolution of centromeric regions in crayfish and other taxa.

SatDNA sequences exhibit great variability in monomer size, nucleotide sequence, genomic distribution and abundance among closely related species [24]. This unique collection of satDNAs distributed in the genome is termed the species’ satellitome. The diversity of satDNA emphasises their rapid evolution while retaining the conserved structural roles in the genome. Divergence of satDNA sequences can be detected at different taxonomic levels, with certain sequences diverging from population to species or higher taxonomic levels [25]. The library hypothesis predicts that related species share a common library of satDNAs inherited from a common ancestor, with differences primarily being quantitative due to differential amplification of certain variants [20, 26–28]. This rapid divergence between species is often explained by concerted evolution, a pattern emerging from processes like satDNA amplification and homogenisation that maintains sequence similarity within a species while allowing independent changes to accumulate in different lineages [17, 25].

SatDNA has long been challenging to study due to the limitations of sequencing technologies and bioinformatic tools [29]. Nowadays, low coverage genome skimming data used in combination with assembly free software allows *de novo* repeat identification from short read sequencing data [29, 30]. This approach enables comprehensive studies across taxa, particularly for highly abundant repeats such as satDNA, even in species with large genomes whose genome assemblies are often still missing [31]. Despite their biological importance, the evolutionary dynamics of satDNA, and their potential impact on intra- and interspecific diversification remain to be fully explored across many taxa [32].

This study aims to elucidate the characteristics and distribution of repetitive DNA, specifically satDNA, across 19 species belonging to four freshwater crayfish families: Astacidae, Cambaridae, Cambaroididae, and Parastacidae. Previous research has highlighted large genome sizes with a high proportion of REs, including satDNA, for several crayfish species [13, 15]. Given this previously observed abundance of satDNA in crayfish, and the known rapid evolution of these sequences in other taxa, we hypothesise that satDNA abundance and diversity vary among freshwater crayfish families, with diversification of satDNA reflecting family-specific evolutionary events. Based on their phylogeographic and evolutionary origins, we further hypothesise that satDNA composition will differ between Northern hemisphere families (Astacidae, Cambaridae, Cambaroididae) and Southern hemisphere families (Parastacidae), allowing us to differentiate these lineages based on their satellitome profiles. Through comparative analyses, we aim to identify conserved and lineage specific repeat sequences and investigate the relationship between the phylogenetic structure and satellitome composition of the species. Understanding the composition and evolution of repetitive DNA in freshwater crayfish is crucial for unravelling the mechanisms that drive genome expansion, structural diversity, and adaptation in this ecologically relevant group.

## Methods

### Sampling and genomic DNA extraction

The male individual of *Astacus astacus* was purchased from the crayfish farm Flusskrebszucht Frömel (Kavelstorf, Germany). The male individual of *Austropotamobius torrentium* was collected from the stream Dolje (Podsused, Croatia) with the permission of the of Croatian Ministry of Economy and Sustainable Development (517-10-1-2-22-4). *Pacifastacus leniusculus* was collected from the river Korana (Croatia) and *Faxonius immunis* from Neuburg am Rhein (Germany). One adult male individual of *Austropotamobius bihariensis* was collected from the Valea Iadului river in Romania (46,7447 N 22,5597 E) with the necessary authorisation from the Romanian Academy (1/CJ/13.01.2021), the Romanian Ministry of Water and Forests (DGB/2/R5787/16.08.2022), the Apuseni Nature Park Administration (199/09.09.2022), the National Agency for Protected Areas (882/15.09.2022), and the Environmental Protection Agencies in the geographical area where the specimen was sampled (76/20.09.2022). All specimens were collected from locations where only a single crayfish species is known to occur, and individuals were examined for morphological characters ensuring reliable species assignment.

Genomic DNA was extracted using a salting out protocol [33] with the following modifications: the digestion of the tissue was performed for 3 h at 65 °C and 400 rpm, to remove proteins and cellular debris the samples were centrifuged at 5000 x g for 10 min, and to precipitate the DNA the samples were centrifuged at 5000 x g for 5 min. Finally, the DNA pellet was resuspended in 100 µL nuclease free water. DNA was quantified using the QuantiFluor^®^ dsDNA System on the Quantus™ Fluorometer (Promega, USA).

### Flow cytometry analysis

The genome size was estimated for *A. astacus* and *A. bihariensis* following a flow cytometry protocol with propidium iodide-stained nuclei [34]. For each species, haemolymph of a − 80 °C adult sample and neural tissue of the internal reference standard *Acheta domesticus* (female, 1 C = 2Gb) was mixed with 2 mL of chopping buffer. Three different buffers were used for different measurements: Galbraith’s buffer [35], Phosphate buffer saline and Otto’s buffer [36]. The suspension was filtered through a 42-µm nylon mesh and stained with the intercalating fluorochrome propidium iodide (PI, Thermo Fisher Scientific) and treated with RNase II A (Sigma-Aldrich), each with a final concentration of 25 µg/mL. The mean red PI fluorescence of stained nuclei was quantified using a CytoFLEX flow cytometer (Beckman-Coulter, USA) with a solid-state laser emitting at 488 nm. Fluorescence intensities of 5000 nuclei per sample were recorded. We used the CytExpert 2.3 software for histogram analyses. The total quantity of DNA in the sample was calculated as the ratio of the mean fluorescence signal of the 2 C peak of the stained nuclei of the crayfish sample divided by the mean fluorescence signal of the 2 C peak of the stained nuclei of the reference standard times the 1 C amount of DNA in the reference standard. The genome size is reported as 1 C. To minimise possible random instrumental errors, three replicates were measured on three different days for each species. The average of these measurements was then used to estimate the genome size 1 C of *A. astacus* and *A. bihariensis*, which is defined as the mean amount of DNA in Gbp in a haploid nucleus.

### Next-generation sequencing

Extracted DNA was fragmented using the Bioruptor^®^ Pico sonication device (Diagenode, Hologic Inc., Liege, Belgium) for 21 cycles of 30 s ON followed by 30 s OFF. Illumina libraries were prepared according to the BEST protocol [37]. Preparation of PCR reactions was automated using a Biomek i7 Hybrid workstation (Beckman Coulter, Brea, CA, USA). Library lengths were verified on a TapeStation system (Agilent Technologies, Santa Clara, CA, USA). Libraries of all species were barcoded, pooled, and sequenced on an Illumina NovaSeq 6000 at Novogene (Cambridge, UK) to obtain 2 × 150 bp paired-end reads. Sequence quality was assessed using FastQC v0.11.9 [38] and quality trimming was performed using the Trimmomatic software [39]. The reads generated during the current study have been deposited in the NCBI SRA repository, BioProjectID PRJNA1293697, sample accession numbers SAMN50031846- SAMN50031850.

### Identification and annotation of repetitive DNA

For the identification of repetitive DNA, the following species were chosen from all crayfish families: *Astacus astacus*, *Austropotamobius torrentium*, *Austropotamobius pallipes*, *Austropotamobius bihariensis*, *Pontastacus leptodactylus*, *Pacifastacus leniusculus*, *Faxonius immunis*, *Faxonius limosus*, *Procambarus clarkii*, *Procambarus acutus*, *Cambarus robustus*, *Cambaroides japonicus*, *Cambaroides dauricus*, *Cambaroides schrenckii*, *Cambaroides similis*, *Cherax destructor*, *Cherax robustus*, *Cherax quadricarinatus*, *Parastacus brasiliensis*. Accession numbers of Illumina paired-end reads obtained in this study and accession numbers of datasets from publicly available studies from the European Nucleotide Archive (ENA) are listed in Supplementary Table 1. Quality control, read pre-processing and RepeatExplorer2 (Galaxy Version 2.3.12.1) analysis were performed following the protocol described in [40]. Reads of each analysed species were filtered against a customised database containing mitochondrial sequences of the species using the RepeatExplorer Utilities: Preprocessing of FASTQ paired-end reads (Galaxy Version 1.0.0.3). The GenBank accession numbers of the mitochondrial sequences used for filtering the reads are listed in Supplementary Table 1. Similarity-based clustering analysis using RepeatExplorer2 [41] was performed using 500,000 reads. The reconstruction of monomer sequences of individual satDNA families was performed using TAREAN analysis [42]. After individual clustering analysis, the reads were concatenated and subjected to comparative analysis using RepeatExplorer2.

### Repeat classification and sequence analysis

Repeat classification was done as follows. After *de novo* identification of repetitive elements in RepeatExplorer2 (Galaxy Version 2.3.12.1), contigs were further classified using Censor [43] and with similarity searches using BLASTN 2.5.0 [44]. and BLASTX 2.5.0 [44]. against public databases (Repbase [45] and NCBI nt database) and against the repetitive elements identified in Rutz et al. (2023) [13]. The sequences were checked for presence of telomeric repeats (TTAGG)_n_. Similarity between the clusters obtained from comparative analysis and individual RepeatExplorer2 runs were analysed using BLASTN 2.5.0. Satellite DNA clusters identified in each species were assigned unique names. Each cluster name consists of: the first letter of the genus name, first two letters of the species, the abbreviation “SAT”, a cluster number corresponding to the output of the RepeatExplorer2 analysis and monomer length in base pairs. Sequences were classified based on length as minisatellites (below 100 bp) and macrosatellites (above 100 bp) [18].

Statistical analyses were conducted in R version 4.2.1 [46]. The normality of GC content and length of satDNA sequences was assessed using the Shapiro Wilk’s test, and the correlation between the two variables was calculated using the Spearman rank correlation test. The distribution of GC content and length of satDNA sequences was assessed using the Wilcoxon test. Comparisons of repeat unit length and GC content among all freshwater crayfish genera and between families was assessed using a Kruskal-Wallis test. Post-hoc pairwise Wilcoxon rank-sum tests with Bonferroni correction were applied to identify specific group differences. For all analyses, the significance level used was α = 0.05.

### Phylogenetic reconstruction and divergence analysis

Phylogenetic reconstruction was based on repeat sequence similarities [47] using a custom bash and R script (Supplementary file 1 and Supplementary file 2). A distance matrix was calculated based on the observed/expected number of edges in clusters between species obtained in comparative analysis using the distance.comb function from the R package sidier v4.1.0 [48]. A dendrogram was built using the R package pvclust v2.2.0 [49] with ward.D2 as the method for hierarchical clustering, Bray–Curtis dissimilarity distance method and 1 000 bootstrap replications. The heatmap was constructed using the R package ComplexHeatmap v2.20.0 [50, 51].

The satDNA consensus sequences obtained from the comparative analysis were used to estimate divergence. First, for all sequences dimers or higher repeat numbers up to 200 nt were generated using the dimerator.py script [52]. This collection was used as a reference for running the RepeatMasker software against reads from each species. RepeatMasker v4.1.2-p1 [53] was used with the parameters -a -nolow -no_is. The sequence divergence distribution was calculated as Kimura distances using the RepeatMasker tool calcDivergenceFromAlign.pl.

### Detailed characterisation of PlSAT3-411 and PlSAT57-664

SatDNAs from each species with similarity to the PlSAT3-411 and PlSAT57-664 were extracted using BLASTN [44] and a consensus sequence was produced using Bowtie2 [54]. For both sequences, dimers were produced using the dimerator.py script [52]. Patterns in the PlSAT3-411 and PlSAT57-664 in all species were examined applying the RepeatProfiler workflow [55, 56]. For the correlation analysis, species were grouped by family. Both consensus sequences were used as reference for the RepeatMasker software against reads from each species.

### Preparation of chromosome spreads and fluorescence *in situ* hybridisation (FISH)

Chromosome spreads were prepared according to the method described in [57]. Specific primers for the satDNA sequence PlSAT3-411 [15] were used for amplification of probes for FISH. PCRs were performed using GoTaq Green Master Mix (Promega, USA) in 25 µL reactions: 12.5 µL GoTaq^®^ Green Master Mix, 2.5 µL of each primer, 1 µL DNA. The PCR program consisted of 3 min denaturation at 95 °C, 35 cycles each with 1 min denaturation at 95 °C, 30 s annealing at 55 °C, 1 min extension at 72 °C, and a final extension of 20 min. Amplicons were visualised on a 2% agarose gel and purified from gel slices using the ReliaPrepTM DNA Clean-Up and Concentration System (Promega, USA). Cloning was performed using a pGEM-T Easy Vector System (Promega, USA) according to the manufacturer’s protocol. Individual clones were purified using the PureYield™ Plasmid Miniprep System (Promega, USA) and sequenced by Macrogen (Amsterdam, The Netherlands).

Plasmid vectors containing the satDNA monomer sequence were labelled with Aminoallyl-dUTP-Cy3 (Jena Bioscience GmbH, Jena, Germany) using the Nick Translation Reagent Kit (Abbott Molecular Inc., USA) according to the manufacturer’s protocol with the following modifications: plasmid DNA (700 ng) was labelled in a reaction volume of 25 µL using 2.5 µL of enzyme mixture for 6 h at 15 °C. FISH was performed according to [15]. The preparations were mounted in Dako Fluorescence Mounting Medium (Dako North America Inc., USA) and stored at 4 °C overnight. Signals were visualised using an Olympus BX51 microscope, equipped with a cooled CCD camera (Olympus DP70).

## Results

### Repeat classification and sequence analysis

**Fig. 1:**
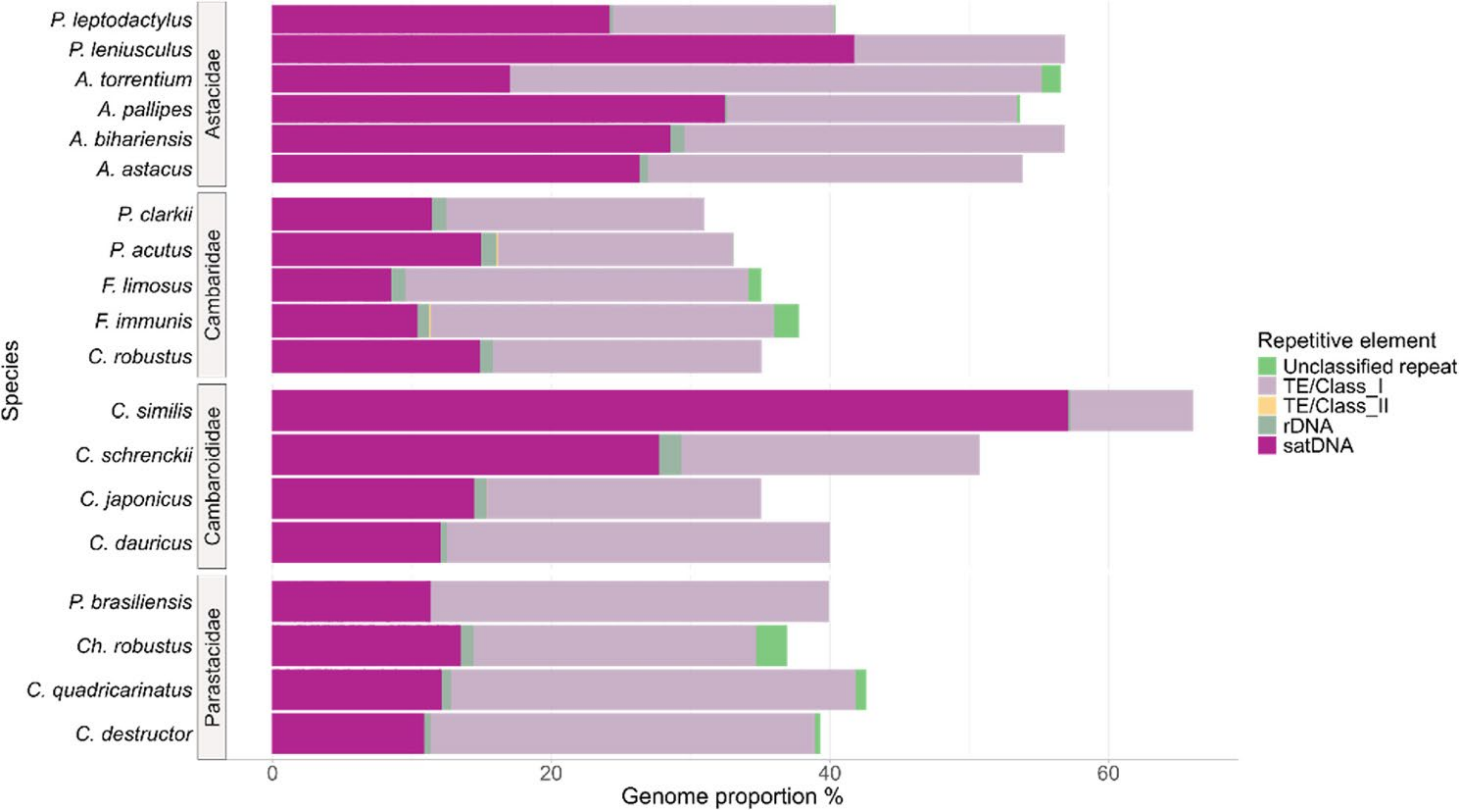
Proportions of repetitive elements in genomic reads of the 19 freshwater crayfish species used in this study. Each bar represents a species, with colours indicating different repetitive element categories. Repetitive elements are annotated as satellite DNA (satDNA), ribosomal DNA (rDNA), TEs belonging to Class I and Class II, and sequences without detailed annotation indicated as Unclassified repeats. Proportions are on a scale from 0 to 65 %

The genome size of *A. astacus* and *A. bihariensis* was measured from haemolymph. Results showed that the average 1 C DNA value was 16.89 Gbp for *A. astacus* and 11.58 Gbp for *A. bihariensis* (Supplementary Table 2). The number of obtained reads per species (*A. torrentium*, *A. bihariensis*, *F. immunis*, *P. leniusculus*, *A. astacus)* resulting from low coverage sequencing ranged from 13.5 M to 40.8 M reads corresponding to 0.15–0.53 x coverage. (Supplementary Table 1). Reads obtained in this study and from public databases were used in the *de novo* identification of repeats in 19 freshwater crayfish species (Supplementary Table 1) with the RepeatExplorer2 pipeline based on low-coverage Illumina reads. The clusters identified by RepeatExplorer2 are shown in Supplementary Table 3. The number of identified clusters ranged between 620 in *A. astacus* and 756 in *P. leptodactylus*. The read proportion of each element, grouped by repeat type, is shown in Fig. 1. All sequences were annotated as satDNA, ribosomal DNA (rDNA), TEs belonging to Class I and Class II, or repeats (sequences without detailed annotation). The total proportion of REs in each species ranged from 30% in *P. clarkii* to 66% in *C. similis*. Ribosomal DNA (rDNA) was identified with 0.08% to 1.5% abundance per species, except for *P. leniusculus*, *A. torrentium* and *P. brasiliensis* where no sequences were assigned to rDNA sequences (Fig. 1, Supplementary Table 3). In each genome, the most abundant REs were either annotated to satDNAs or Class I TEs, which were represented by LINE and LINE/DIRS elements. In the Astacidae family, the highest proportion of reads was represented by satDNA sequences ranging from 17% in *A. torrentium* to 41.7% in *P. leniusculus*. The second highest proportion of reads in the Astacidae family belonged to Class I TEs, while no Class II elements were annotated. In the Cambaridae family, the highest proportion of reads were assigned to Class I TEs (between 16% in *P. acutus* and 24% in both species of the genus *Faxonius*), while 8 to 14% of sequences were assigned to satDNA. Class II TE were assigned to 0.01% (Maverick element) and 0.05% (Helitron element) of the sequences in *F. immunis* and *P. acutus*, respectively. In the Cambaroididae family, most sequences were assigned to Class I TEs in *C. japonicus* and C. *dauricus* with 19% and 27%, respectively, while in *C. similis* and *C. schrenckii* most sequences were assigned to satDNA with 57% and 27%, respectively. In *C. japonicus*, 0.01% of reads were assigned to Class II TE Maverick. In the Parastacidae family, most sequences were assigned to Class I TEs, ranging between 20% in *Ch. robustus* and 29% in *C. quadricarinatus*. Among the studied species, distinct satDNA sequences dominate their respective genomes (Supplementary Table 3, Supplementary Table 4). In genomes with the highest proportion of satDNA, the most abundant satDNA are CsiSAT1-17 in *C. similis* with 38% of reads assigned, PleSAT1-21 in *P. leniusculus* with 22% of reads assigned and ApaSAT1-21 in *A. pallipes*.

**Fig. 2:**
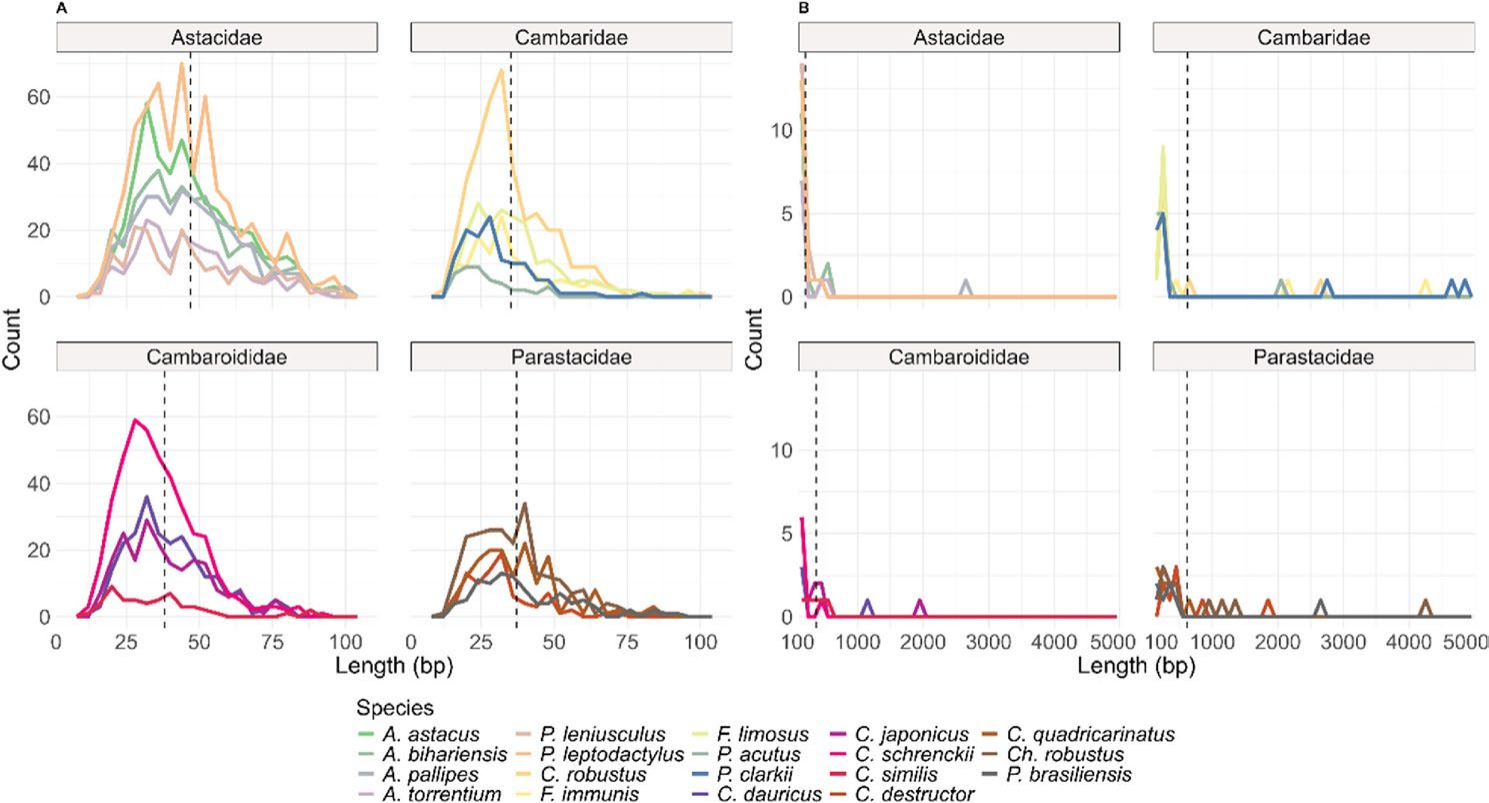
Frequency of satDNA sequences length across studied species. **A** Minisatellite DNA sequences ≤ 100 bp. **B** Macrosatellite DNA sequences > 100 bp. Each colour represents a species. Vertical dashed lines represent the mean length value for each family

The range of the repeat unit length of satDNA sequences was from 12 to 2657 bp for the Astacidae family, from 14 to 4898 bp for the Cambaridae family, from 12 to 1920 bp for the Cambaroididae family and 13 to 2700 bp for the Parastacidae family (Supplementary Table 3). The distribution of the repeat unit length of satDNA sequences is concentrated below 100 bp (Fig. 2). The mean repeat unit length for minisatellite sequences (below 100 bp) were 46.9 bp for Astacidae, 35.2 bp for Cambaridae, 38.1 bp for Cambaroididae and 37.1 bp for Parastacidae. The mean repeat unit length for macrosatellite sequences (above 100 bp) were 201.7 bp for Astacidae, 622.1 bp for Cambaridae, 368.3 bp for Cambaroididae and 608.5 bp for Parastacidae (Fig. 2). Overall, the length of repeat unit sequences of satellites was significantly different between genera (Kruskal-Wallis test, *p* = 5.157e-68) and between families (Kruskal-Wallis tests, *p* = 2.537e-70) (Supplementary Table 5). From now on, we will refer to satDNA sequences below 100 bp as minisatellites, satDNA sequences above 100 bp as macrosatellites, and all satDNA as simply satellites [18].

**Fig. 3:**
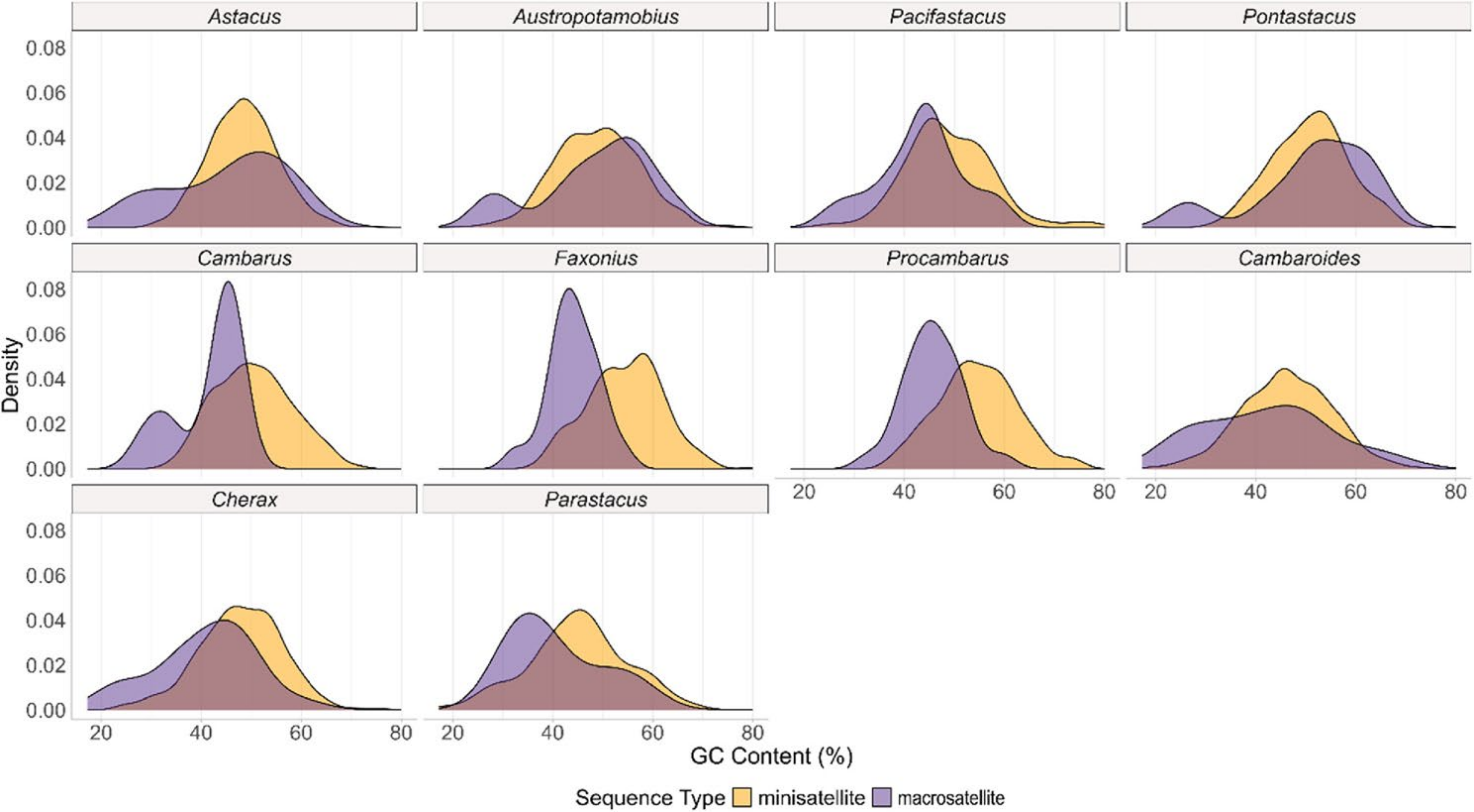
Density plot representing the GC content distribution for minisatellite and macrosatellite sequences by crayfish genus. Colours represent sequence type minisatellite (< 100 bp) and macrosatellite (> 100 bp)

The GC content of the satDNA sequences across all studied species varied between 17.2% (PbrSAT14-29 in *P. brasiliensis*) and 80% (FimSAT163-15 in *F. immunis*). The median GC content value for minisatellites was 49.19% and 46.15% for macrosatellites (Supplementary Table 3). The GC content differed significantly between macrosatellites and minisatellites (Wilcoxon rank-sum test, *p* = 1.048e-05) (Fig. 3). We observed two different populations of macrosatellites in terms of GC content in the genera *Astacus*, *Austropotamobius*, *Pacifastacus*, *Pontastacus* and *Cambarus*: one peak of satDNA distributed at around 30% GC content and a second peak at 50% GC content (Fig. 3). GC content was different for all satDNA between genera (Kruskal-Wallis test, *p* = 5.252 e-67) and between families (Kruskal-Wallis test, *p* = 1.0156 e-56) (Supplementary Table 5). A negative correlation was observed between GC content and repeat unit length overall in satellite DNA (Spearman’s rho = -0.056, *p* = 1.581 e-04) and in minisatellites (Spearman’s rho = -0.035, *p* = 0.0205). On family level, a negative correlation was observed only for the family Astacidae and Cambaridae for satDNA (Supplementary Fig. 1, Supplementary Table 6).

### Phylogenetic reconstruction and divergence analysis

Hierarchical clustering was performed based on the clusters obtained from comparative analysis of all species with RepeatExplorer2. Based on the cluster similarities, two clusters were observed: one for species of the family Parastacidae, and another for all the other freshwater crayfish families. Species belonging to the same family clustered together except for *F. limosus* and *P. clarkii* (family Cambaridae) that grouped together with the species of the family Astacidae (Supplementary Fig. 2).

**Fig. 4:**
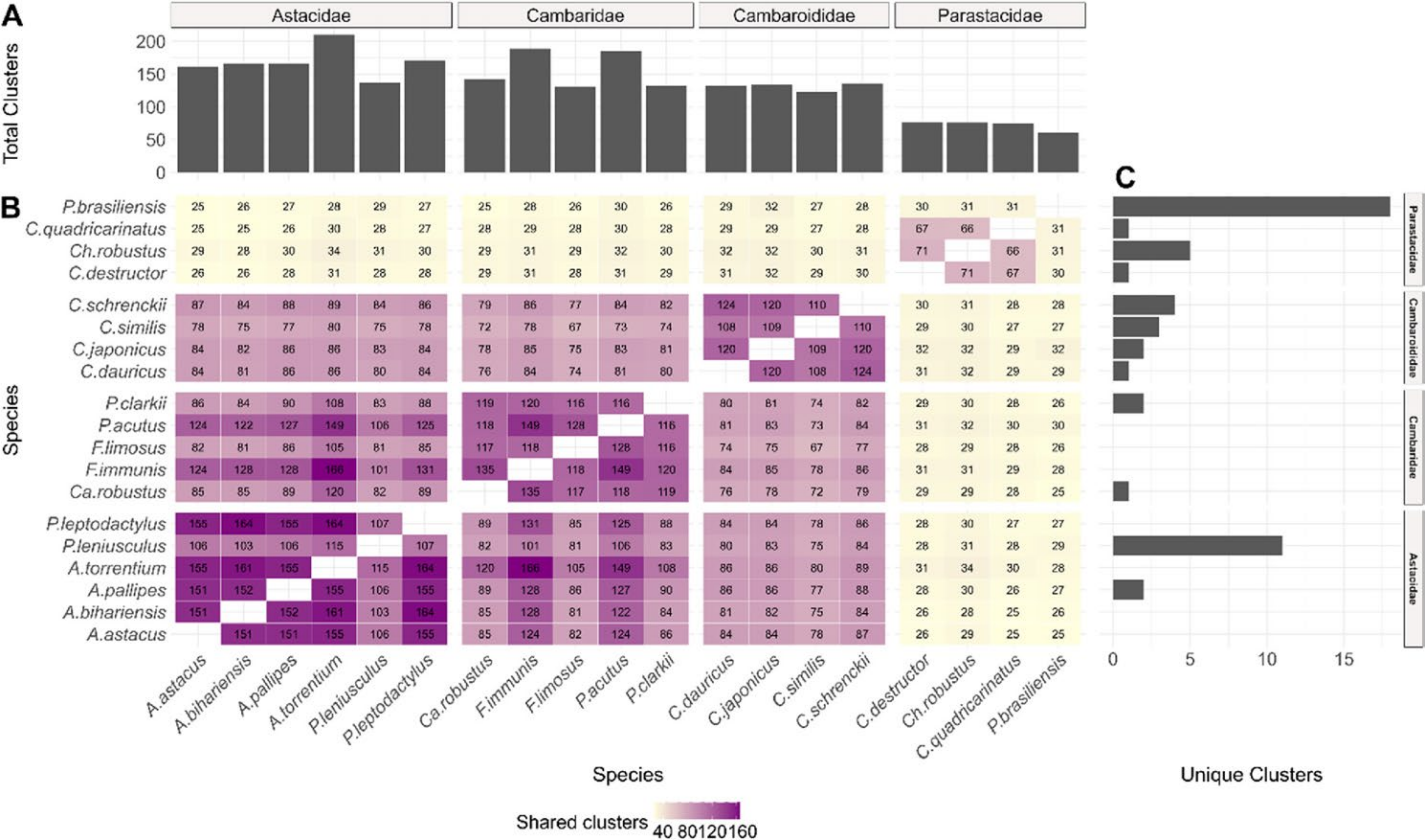
**A** Total number of RE clusters per species identified in comparative analysis by Repeat Explorer2. **B** Number of share clusters between species. **C** Number of unique clusters per species

Based on comparative analysis, we identified in total 395 RE clusters (Fig. 4). The highest number of RE clusters were present in the species *A. torrentium* and the lowest in *P. brasiliensis* (Fig. 4.A). Most of the identified clusters appeared in the species within the family Astacidae, while the lowest number of shared RE clusters was present between the species of the family Parastacidae and all other species (Fig. 4.B). The number of unique clusters per species was generally low (< 5), except for *P. brasiliensis and P. leniusculus* with 18 and 11 unique clusters respectively (Fig. 4.C).

**Fig. 5:**
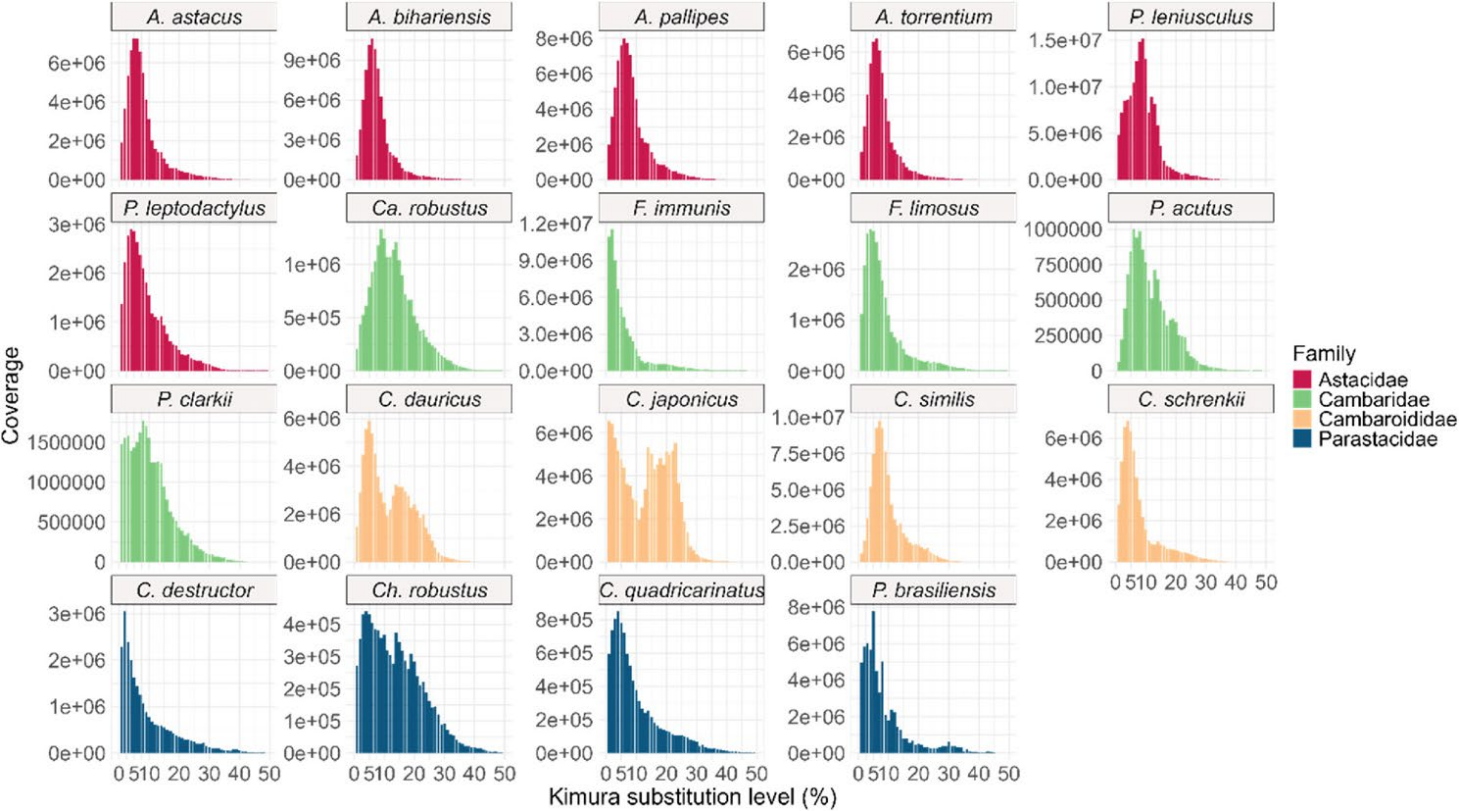
SatDNA sequence divergence landscapes for each species. Colours indicate different freshwater crayfish families. y-axes are scaled for each species

Sequence divergence landscapes were calculated for all satDNA sequences in each species. The analysis revealed distinct patterns across the crayfish species and families (Fig. 5). In the family Astacidae, a peak was observed at lower substitution levels (0–10%). In Cambaridae, distinct patterns were observed between species of the genus *Faxonius* (one peak at 0–10%), the genus *Procambarus* (multiple peaks between 0 and 20%), and *Ca. robustus* (peak between 5 and 20%). In the family Cambaroididae, distinct patterns were observed between *C. similis and C. schrenckii* (one peak at 0–10%), and between *C. japonicus* and *C. dauricus* (one peak at 0–10% and another peak at 20–30%). In *Ch. robustus*, multiple peaks were present at 0–30% divergence, while the other species in the family Parastacidae show one peak at lower substitution levels (0–10%). The overall satDNA content varies between species as indicated by the different coverage (Fig. 5).

### Detailed characterisation of PlSAT3-411 and PlSAT57-664

**Fig. 6:**
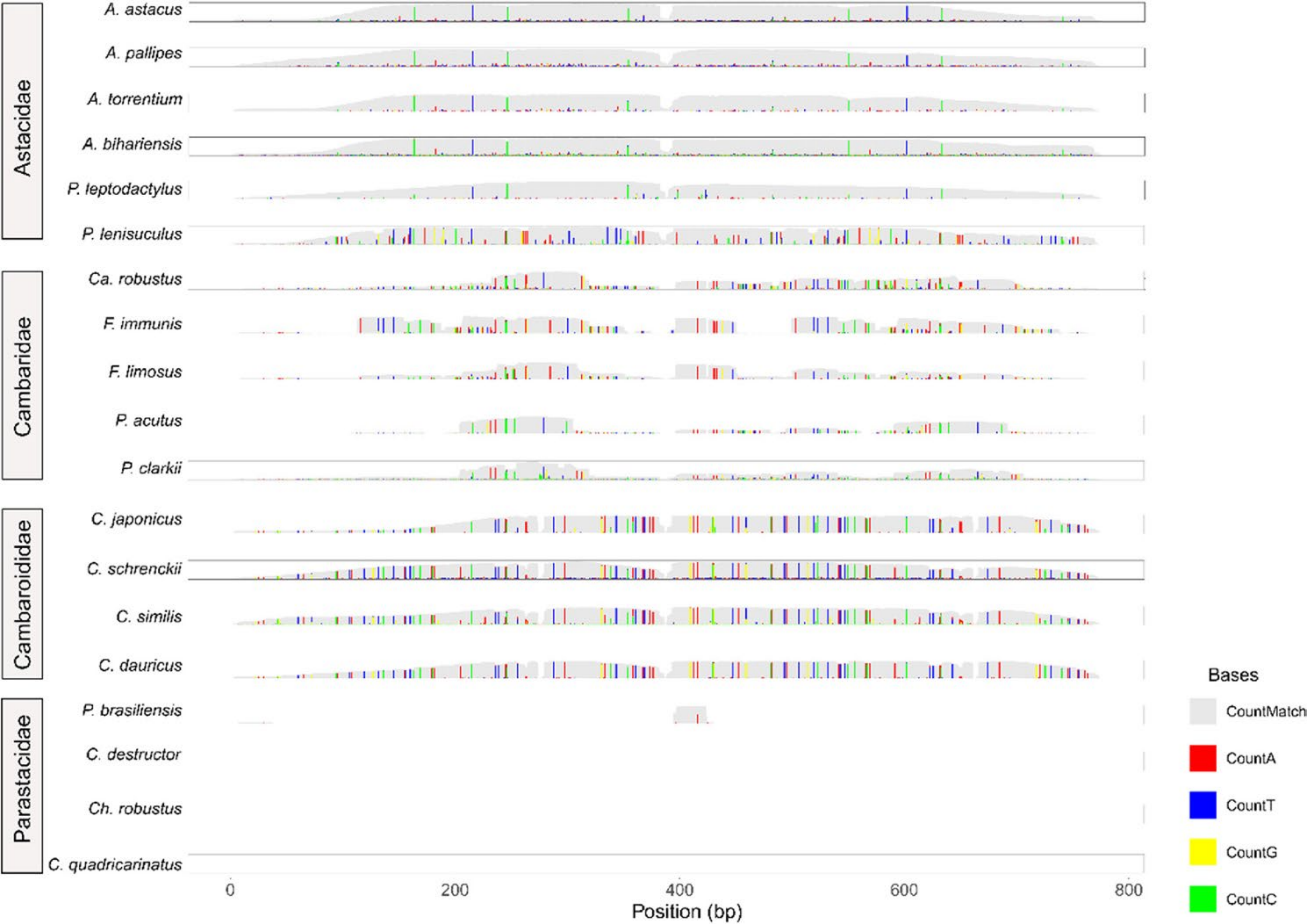
Variant repeat profiles of PlSAT3-411 satellite DNA family across the studied species. Different colours indicate A, T, C and G bases. The height of each bar indicates the coverage of each variant in the reads

The PlSAT3-411 satDNA family was previously identified as pericentromeric satellite DNA in *P. leptodactylus* [15] and was selected for further analysis. Dimer consensus sequences of the PlSAT3-411 sequence were used to obtain repeat profiles of the sequence in each species. The colour enhanced profiles showed the presence of the entire sequence in the species from the Astacidae and Cambaroididae family with similar depth (Supplementary Fig. 3). In the Cambaridae family, only parts of the sequence were mapped, while the sequence is not present in the Parastacidae family. Variant profiles show family-specific signatures in the pattern of variants relative to the consensus PlSAT3-411 sequence (Fig. 6). Based on the analysis of the PlSAT57-664 sequence, we observed the largest depth for the species *C. robustus*, *F. immunis* and *F. limosus*and partial mapping to the reads in most of the species (Supplementary Fig. 4.).

**Fig. 7:**
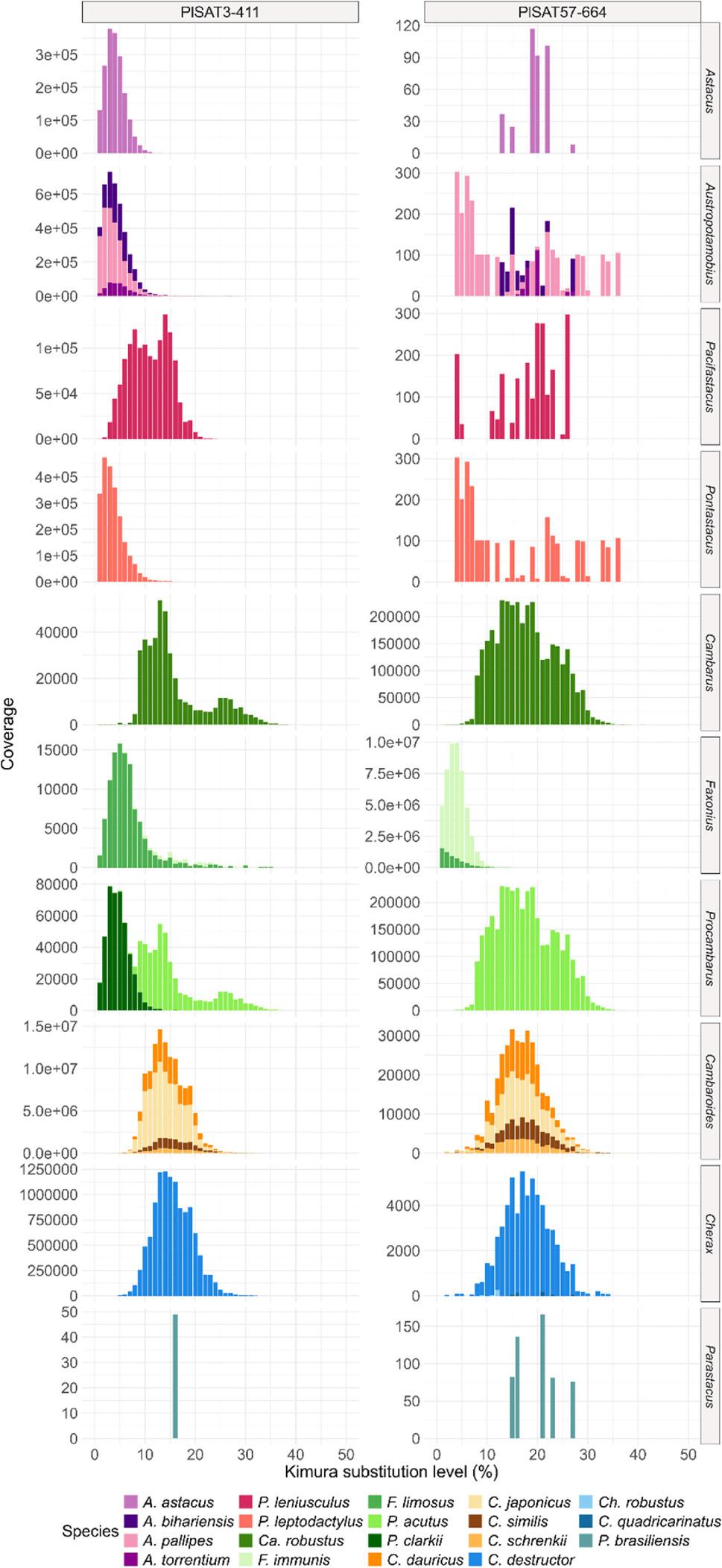
Sequence divergence landscapes for each species for the PlSAT3-411 (left) and PlSAT57-664 (right) sequences. Colours indicate different species. y-axis is scaled for each species

The distribution of Kimura substitution levels was explored for the PlSAT3-411 and PlSAT57-664 sequences in each species. The landscapes varied among species and between the two satellite sequences. In the Astacidae and Cambaroididae family, the PlSAT3-411 sequence showed high coverage and a peak at lower substitution levels (0–5%). In contrast, the PlSAT57-664 sequence showed 10^5-fold lower coverage than the PlSAT3-411 sequence. The pattern was opposite for the species in the Cambaridae family, with the PlSAT57-664 sequence having a double coverage compared to the PlSAT3-411 in all genera, except in the genus *Faxonius* where the coverage of the PlSAT57-664 sequence was 10^5-fold higher than the PlSAT3-411 sequence. In the genera *Cambarus* and *Procambarus* (Cambaridae family) the divergence peak was present at 5–10% for the PlSAT3-411 sequence and 10–30% for the PlSAT57-664 sequence. In the genus *Faxonius*, the PlSAT57-664 showed a peak at 0–10% divergence with higher coverage for *F. immunis* than *F. limosus* (Fig. 7). To check for the chromosomal localisation of the PlSAT3-411, FISH was performed on metaphase spreads of *A. torrentium* and *P. leniusculus*. The chromosome mapping revealed hybridisation sites in the pericentromeric regions on all chromosomes in both species (Supplementary Fig. 5.).

## Discussion

We investigated the repetitive genomic landscape of freshwater crayfish to gain novel understanding of the genome evolution through satellite DNA in 19 species across four families. In line with our hypothesis, our findings highlight universal patterns present across-freshwater crayfish families, as well as lineage specific sequences, providing unique insights into repetitive element diversification in freshwater crayfish. The relevance of our results regarding our research hypothesis is discussed below.

### High number of satellite DNA families in all freshwater crayfish species

The high number of REs identified across all studied species aligns with previous findings in Decapoda, where the proportion of REs constitutes 58% to 79% of genomes [13]. Our results confirm an overall high repetitive DNA content in all species, ranging from 30% in *P. clarkii* to 66% in *C. similis* (Fig. 1). We observed family-specific patterns in the distribution of transposable elements and satDNA, with Astacidae and Cambaroididae showing the highest proportion of satDNA, while Cambaridae and Parastacidae genomes were dominated by Class I transposable elements (Fig. 1). The lack or low number of identified Class II TEs observed here has already been reported in other Decapoda [13, 58]. Class I TEs tend to accumulate in low-recombination regions such as peri-centromeric heterochromatin, while Class II are more common in gene rich regions [59]. The “copy and paste” proliferation mechanism of Class I TE can lead to TE copy accumulation, contributing to larger genome sizes [59]. The prevalence of TEs has been identified as a key factor underlying genome size variation in Decapoda [13, 60].

Eukaryotes exhibit a wide array of satDNA families, ranging from 2 in the plant *Tanacetum cinerariifolium* [61] to 226 in the frog *Proceratophrys boiei* [62] and 258 in *P. leptodactylus* [15]. Even within genera, satDNA counts can vary greatly, as seen in the beetle *Tribolium freemani* with 135 satDNA families and *T. castaneum* with 57 satDNA [27, 63]. In our study, the number of satDNA families varies greatly, but is overall high, between 54 and 622 (Supplementary Table 3). This illustrates the remarkable diversification of the satellitome in freshwater crayfish. A high proportion of satDNA in all species in this study is formed by minisatellites (< 100 bp), especially sequences shorter than 50 bp (Fig. 2). A high abundance of minisatellites has also been found in other Decapoda genomes [64, 65]. Minisatellites are often associated with euchromatic regions [18, 66], but they can also be associated with long arrays present in pericentromeric and subtelomeric regions [18]. In *P. leptodactylus*, the minisatellites PlSAT6-70 and PlSAT14-79 are located interstitially along the chromosome arms, considered part of euchromatic regions [15]. The formation of new minisatellite sequences arises from DNA replication, DNA recombination or DNA repair [67]. Some minisatellites found in euchromatic regions or within genes appear to be essential for protein function [67]. Furthermore, it has been proposed that minisatellite sequences facilitate interchromosomal DNA exchanges [68, 69]. Overall, these findings suggest the large number of minisatellites form a highly dynamic component of crayfish genomes, contributing to genome diversification.

### SatDNAs among freshwater crayfish show shared characteristics

Our study revealed distinct satDNA sequence characteristics across the 19 species belonging to the four extant crayfish families, including variations in repeat unit length, GC content (Supplementary Table 3), and substitution landscapes (Fig. 5). We observed a negative correlation between GC content and satellite length overall for satellites and minisatellites (Fig. 3, Supplementary Fig. 1). A negative correlation has been found in the grasshopper *Locusta migratoria* and in the fish *Astyanax paranae*, where shorter satDNA tend to arise from GC-rich regions, while longer monomers are more AT-rich [70, 71]. Other studies in fish genera *Megaloporinus*, *Psalidodon* and *Astyanax* did not establish correlation between these two characteristics [72, 73]. It is generally considered that AT-rich satDNA can induce DNA curvature, important for facilitating the tight packing of DNA and proteins in heterochromatin [74]. Heterochromatin acts as a scaffold for nuclear architecture, enabling the efficient organisation and proper chromosome segregation during mitosis and meiosis [75], therefore ensuring the proper organisation of the large number of chromosomes.

### Phylogenetic signal of satDNA

Among the analysed freshwater crayfish families, species of the family Parastacidae shows the highest number of unique clusters (Fig. 4). Hierarchical clustering based on the repeat profiles separated the family Parastacidae from the other families (Supplementary Fig. 2), consistent with their distinct biogeographic history and deep phylogenetic divergence from Northern Hemisphere crayfish [1]. The clustering of the three other families into a single group reflects higher repeat similarity, despite their taxonomic diversity. While hierarchical clustering of satDNA profiles aligns with the divergence of Parastacidae, the clustering of the other crayfish families suggests that satDNA evolution is also influenced by lineage-specific homogenisation processes within the whole superfamily Astacoidea. Repeatome based analyses have revealed clear phylogenetic signals in different plant groups [47, 76], and several studies showed the use of single satDNA as phylogenetic markers [77, 78]. The clustering of species in our study based on satDNA does not fully mirror their taxonomy. Stronger phylogenetic signals could arise by performing clustering analysis at the family or genus level or by focusing only on a smaller number of satDNA sequences. Clustering can be further influenced by high intraspecific diversity and the presence of multiple divergent satDNA subfamilies. Low sequence divergence peaks in the family Astacidae and in two Cambaroididae species, *C. similis* and *C. schrenckii* (0–10%, Fig. 5) suggest the rapid spread and amplification of similar repeat units within a species, resulting in a high abundance of recently homogenised sequences that have not yet accumulated many mutations [79, 80]. This could reflect rapid and localised speciation events or rapid population increases potentially driven by paleohydrogeological changes. Conversely, multiple and broader peaks present in *Ca. robustus*, *P. acutus*, *Ch. robustus*, *C. dauricus* and *C. japonicus* (Fig. 5) indicate that different subfamilies within a satDNA family are diverging independently, thus creating more sequence variants. This pattern suggests older and more complex speciation histories which aligns with the wide distribution and large number of species present in the families Parastacidae and Cambaridae or reflects distinct diversification events within the *Cambaroides* genus.

### Conserved satDNAs at the core of the freshwater crayfish satellitome

The presence of certain satDNA sequences with low sequence divergence (as seen in the PlSAT3-411, Figs. 6 and 7) can be attributed to the concerted evolution and strong purifying selection in functionally constrained genomic regions [81]. SatDNA sequences found near important functional regions such as ribosomal loci or within euchromatic regions can exhibit minimal divergence due to functional constraints and potentially deleterious effects of mutations [81, 82]. The distribution of the PlSAT3-411 sequence throughout all the species aligns with patterns of concerted evolution, where the repetitive DNA sequences maintain a greater similarity among repeats within a species than between species [18, 24]. This conservation may reflect functional roles in centromeric/pericentromeric regions, while its absence in Parastacidae suggests deep evolutionary divergence. Although our results suggest an association of the PlSAT3-411 sequence with centromeric regions, future FISH analyses should be extended to a broader range of species. Furthermore, the localisation should be validated using complementary methods, such as immunostaining and immunoprecipitation using centromere specific protein or long read sequencing. Due to abundance of PlSAT57-664 in the family Parastacidae and its sequence similarity to PlSAT3-411, we can speculate that PlSAT57-664 family may have a similar role across these species, however, this hypothesis should be confirmed in future by chromosomal localisation studies. However, the observed lack of similarity of the PlSAT57-664 between species could be due to the high divergence of species-specific monomers, thus influencing the structural role of this sequence family. The functional roles of centromeric satDNA include contributing to structural integrity of the centromere [83], maintenance of the heterochromatin, epigenetic regulation through non-coding RNAs [84], recruitment of centromere specific proteins during cell replications and maintenance of genome stability [20]. High AT content and canonical size of 170–340 bp have been proposed to support the packaging of DNA in the heterochromatin, providing crucial satDNA function typical for centromeric satellites [74]. The functional role of the PlSAT3-411 family, as pericentromeric satDNA, has already been hypothesised in [15]. The contrasting abundance of PlSAT3-411 and PlSAT57-664 among the freshwater crayfish families parallels the library hypothesis of satDNA evolution, which suggests that related species share a common library of satDNA sequences inherited from the last common ancestor but differentially amplified across closely related lineages. This process leads to species-specific satDNA profiles, with similar sequences in different proportions observed among the species with a common ancestor [17, 26]. While satDNA is usually highly dynamic, several studies have reported conservation over periods longer than 50 Mya [85]. In molluscs, two satDNAs, BIV160 and PjHaaI, have been discovered to be conserved for 540 Mya [86, 87]. The divergence between Northern and Southern Hemisphere crayfish families (Parastacidae vs. Astacidae, Cambaridae, Cambaroididae) occurred around 241 Mya [4], suggesting that the PlSAT3-411 is at least 241 Mya old. This makes the PlSAT3-411 one of the most ancient satDNAs discovered so far.

### The challenges of studying satellitomes of non-model organisms

The study of REs, especially satDNA, across diverse organisms poses computational challenges due to the long, highly repetitive nature of these sequences, which complicates genome assembly and bioinformatic annotation [88]. Consequently, satDNA is often underrepresented in databases, and most satellitome studies are still often performed on one or a few species. Here we focused on species representative of all four freshwater crayfish families, capturing diversity across genera and highlighting lineage specific satDNA trends. Low coverage genome sequencing circumvents the challenges associated with assembling highly repetitive regions in large, complex genomes. This approach has been demonstrated to provide reliable repeat profiles across a range of diverse taxa [31]. Although this approach effectively identifies satDNA, it has limitations for the characterisation of other repeat classes, such as TE which often require long read sequencing. By integrating *de novo* tools such as RepeatExplorer and TAREAN and analysing metrics like monomer clustering and GC content we provide an approach to understanding crayfish genome evolution.

## Conclusion

Here we provide novel insights into the repetitive DNA and highlight conserved and lineage-specific patterns of satDNA evolution across 19 freshwater crayfish species from four families. Our results reveal a high number of satDNA families and a high proportion of REs in all species, confirming the important role of repetitive DNA in shaping freshwater crayfish genomes. Our results show distinct family-level patterns, with Parastacidae exhibiting the highest number of unique repeat clusters and phylogenetic separation from Northern Hemisphere families, consistent with their evolutionary divergence. The observed variation in satDNA repeat unit length, GC content, and substitution landscapes reveals dynamic processes, including concerted evolution and differential amplification, as predicted by the library hypothesis. The conservation of certain satDNAs across all species highlights their likely functional roles, particularly in centromeric or pericentromeric regions. This study advances our understanding of genome evolution in decapods and emphasises the value of satDNA in unravelling both evolutionary history and functional genome organisation in crustaceans.

## Supplementary Information

Supplementary Material 1: Supplementary figure 1. Correlation of GC content (%) and repeat unit length (bp) for (A) overall satellites (B) overall satellites per family, (C) minisatellites and (D) minisatellite per family. Colours indicate different genera. Correlation was tested using Spearman rank correlation test with significance level α=0.05. Significance levels are indicated as follows: p < 0.05 *, p < 0.01 **, and p < 0.001 ***.Supplementary figure 2. (A) Cluster dendrogram and (B) heatmap showing hierarchical clustering of satDNA sequences in all 19 species based on observed/expected number of edges between species in RepeatExplorer2 analysis. In (A) red numbers on nodes indicate Approximately Unbiased (AU) p-value, while green numbers on nodes indicate Bootstrap Probability (BP) values. Clusters with AU larger than 95% are highlighted by rectangles. In (B) colours indicate distance values. Supplementary figure 3. Colour enhanced profile of PlSAT3-411 satellite DNA family against each species. Different colours indicate coverage. The height of each bar indicates the coverage of base variant in the readsSupplementary figure 4. Variant repeat profiles of PlSAT57-664 satellite DNA family across the studied species. Different colours indicate A, T, C and G bases. The height of each bar indicates the coverage of each variant in the reads.Supplementary figure 5. Localisation of PlSAT3-411 satellite repeat family (in red) on metaphase chromosomes of (A) *A. torrentium* and (B) *P. leniusculus*. Red signals represent the Cy3-labeled probe localisation, chromosomes are counterstained with DAPI. Scale bar = 10μm.Supplementary table 1. Overview of the analysed species, including their genus, family, accession number of the reads, number of sequencing reads obtained in this study, mitochondrial genome accession number and genome size (Gb) Supplementary table 2. Flow cytometry genome size measurement of haemolymph from *Astacus astacus* and *Austropotamobius bihariensis* obtained by PI fluorescence dye excitation with three chopping buffers.Supplementary table 3. Summary of clusters identified in individual RepeatExplorer runs for each crayfish species. For each cluster are indicated the unique cluster name (CL_unique), the supercluster classification, the cluster size, automatic annotation from RepeatExplorer, TAREAN annotation, the final manually curated annotation and genome proportion (%). For satDNA sequences the sequence, length (bp) and GC content (%) are indicated.Supplementary table 4. Summary of clusters identified in the comparative RepeatExplorer run. For each cluster are indicated RepeatExplorer classification, TAREAN classification, total number of reads in a cluster and number of reads in a cluster belonging to a particular species, and correspondence to satDNA in individual clustering. Supplementary table 5. Summary of Kruskal-Wallis tests comparing GC content and satDNA repeat length across genera and families. The table reports the tested variable, grouping factor, test statistic (Chi-squared), degrees of freedom (Df), and corresponding p-value for each comparison.Supplementary table 6. Spearman rank correlation test between GC content and satDNA repeat length across freshwater crayfish families. The table reports the correlation value (Spearman’s rho) and corresponding p-value for each family.Supplementary table 7. Results of pairwise Wilcoxon rank-sum tests comparing GC content and satDNA repeat length across genera and families. Adjusted p-values were calculated using the Bonferroni correction to control for multiple comparisons. Significance levels are indicated as follows: p < 0.05 *, p < 0.01 **, and p < 0.001 ***. The table includes the tested variable, grouping factor, compared groups, adjusted p-values, and significance annotations.Supplementary file 1. Cluster_similarity.shSupplementary file 2. Cluster_similarity.R.

## Data Availability

The reads generated during the current study have been deposited in the NCBI SRA repository, BioProjectID PRJNA1293697, sample accession numbers SAMN50031846- SAMN50031850.
