# Supplementary material for: The extraordinary satellitome diversity of freshwater crayfish: a driver of genome evolution
Source: Mob DNA. 2026 Apr 28;17:12. doi: 10.1186/s13100-026-00399-8 (PMC13130569; doi:10.1186/s13100-026-00399-8)
Supplement: Supplementary file 1 — Supplementary Material 1: Supplementary figure 1. Correlation of GC content (%) and repeat unit length (bp) for (A) overall satellites (B) overall satellites per family, (C) minisatellites and (D) minisatellite per family. Colours indicate different genera. Correlation was tested using Spearman rank correlation test with significance level α=0.05. Significance levels are indicated as follows: p < 0.05 *, p < 0.01 **, and p < 0.001 ***.Supplementary figure 2. (A) Cluster dendrogram and (B) heatmap showing hierarchical clustering of satDNA sequences in all 19 species based on observed/expected number of edges between species in RepeatExplorer2 analysis. In (A) red numbers on nodes indicate Approximately Unbiased (AU) p-value, while green numbers on nodes indicate Bootstrap Probability (BP) values. Clusters with AU larger than 95% are highlighted by rectangles. In (B) colours indicate distance values. Supplementary figure 3. Colour enhanced profile of PlSAT3-411 satellite DNA family against each species. Different colours indicate coverage. The height of each bar indicates the coverage of base variant in the readsSupplementary figure 4. Variant repeat profiles of PlSAT57-664 satellite DNA family across the studied species. Different colours indicate A, T, C and G bases. The height of each bar indicates the coverage of each variant in the reads.Supplementary figure 5. Localisation of PlSAT3-411 satellite repeat family (in red) on metaphase chromosomes of (A) A. torrentium and (B) P. leniusculus. Red signals represent the Cy3-labeled probe localisation, chromosomes are counterstained with DAPI. Scale bar = 10μm.Supplementary table 1. Overview of the analysed species, including their genus, family, accession number of the reads, number of sequencing reads obtained in this study, mitochondrial genome accession number and genome size (Gb) Supplementary table 2. Flow cytometry genome size measurement of haemolymph from Astacus astacus and Austropotamobius bihariensis obtaine [file 13100_2026_399_MOESM1_ESM.zip › Supplementary material/Supplementary material.docx]

# Supplementary material


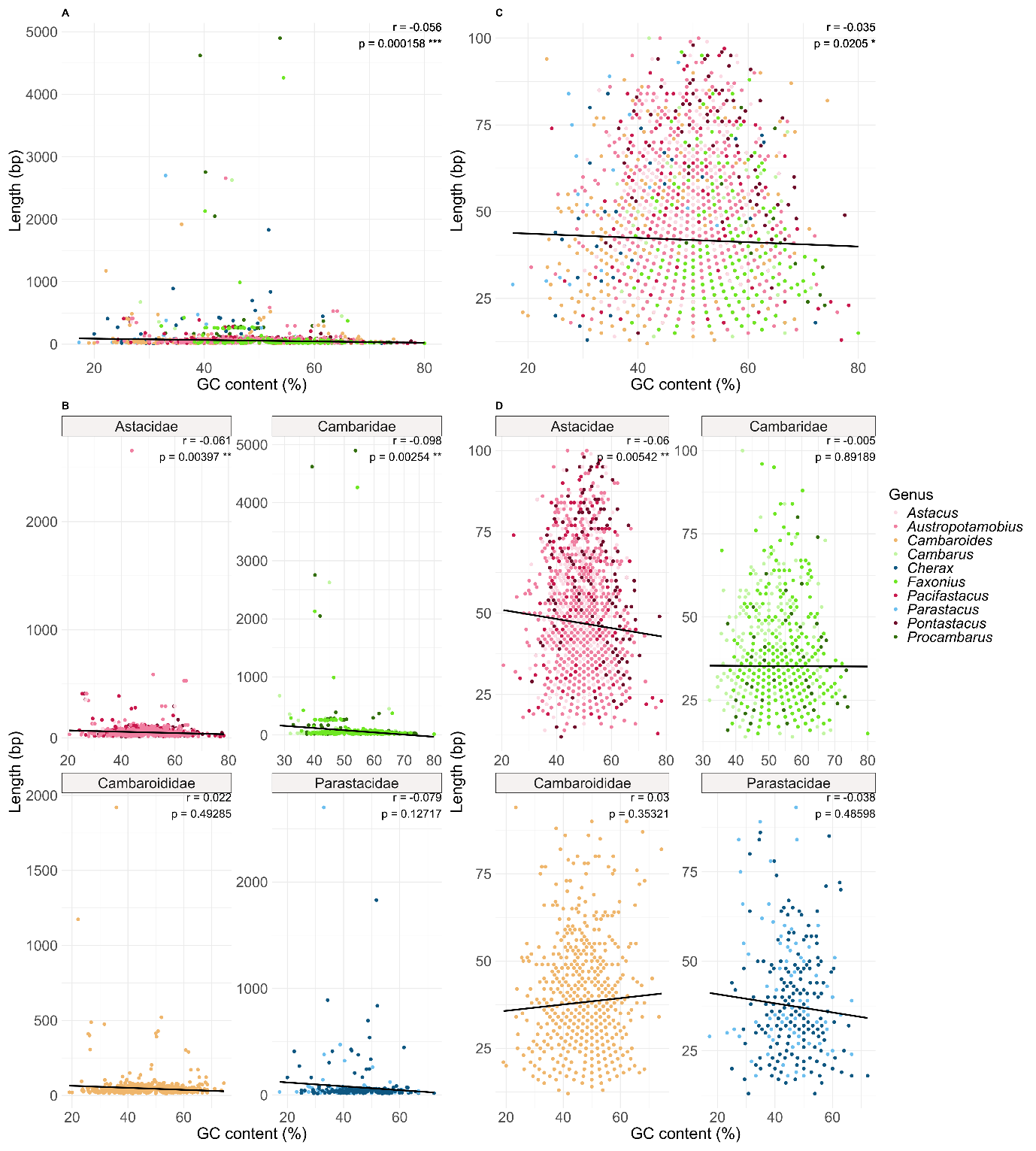


Supplementary figure 1. Correlation of GC content (%) and repeat unit length (bp) for (A) overall satellites (B) overall satellites per family, (C) minisatellites and (D) minisatellite per family. Colours indicate different genera. Correlation was tested using Spearman rank correlation test with significance level α=0.05. Significance levels are indicated as follows: p < 0.05 *, p < 0.01 **, and p < 0.001 ***.


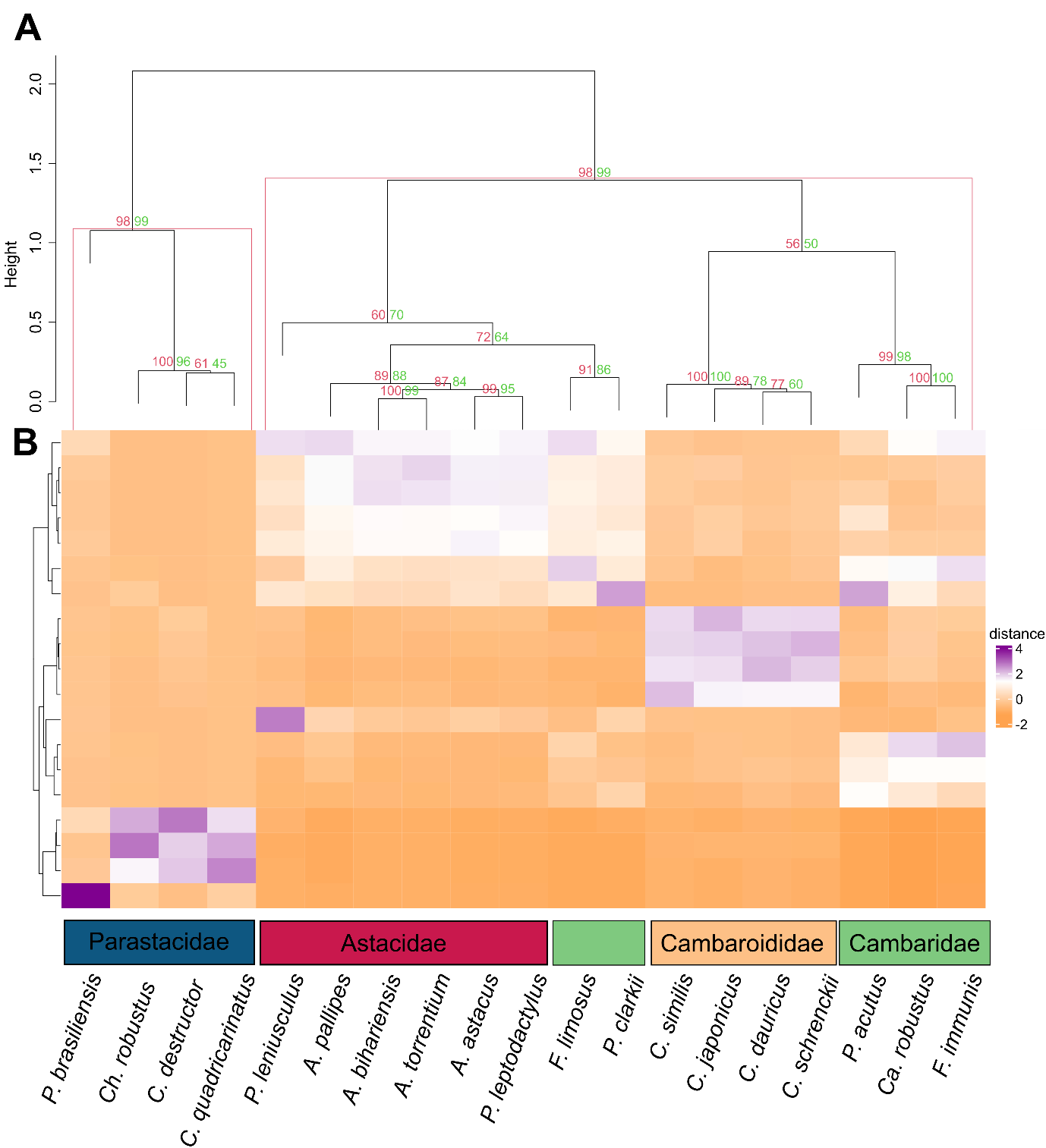


Supplementary figure 2. (A) Cluster dendrogram and (B) heatmap showing hierarchical clustering of satDNA sequences in all 19 species based on observed/expected number of edges between species in RepeatExplorer2 analysis. In (A) red numbers on nodes indicate Approximately Unbiased (AU) p-value, while green numbers on nodes indicate Bootstrap Probability (BP) values. Clusters with AU larger than 95% are highlighted by rectangles. In (B) colours indicate distance values.


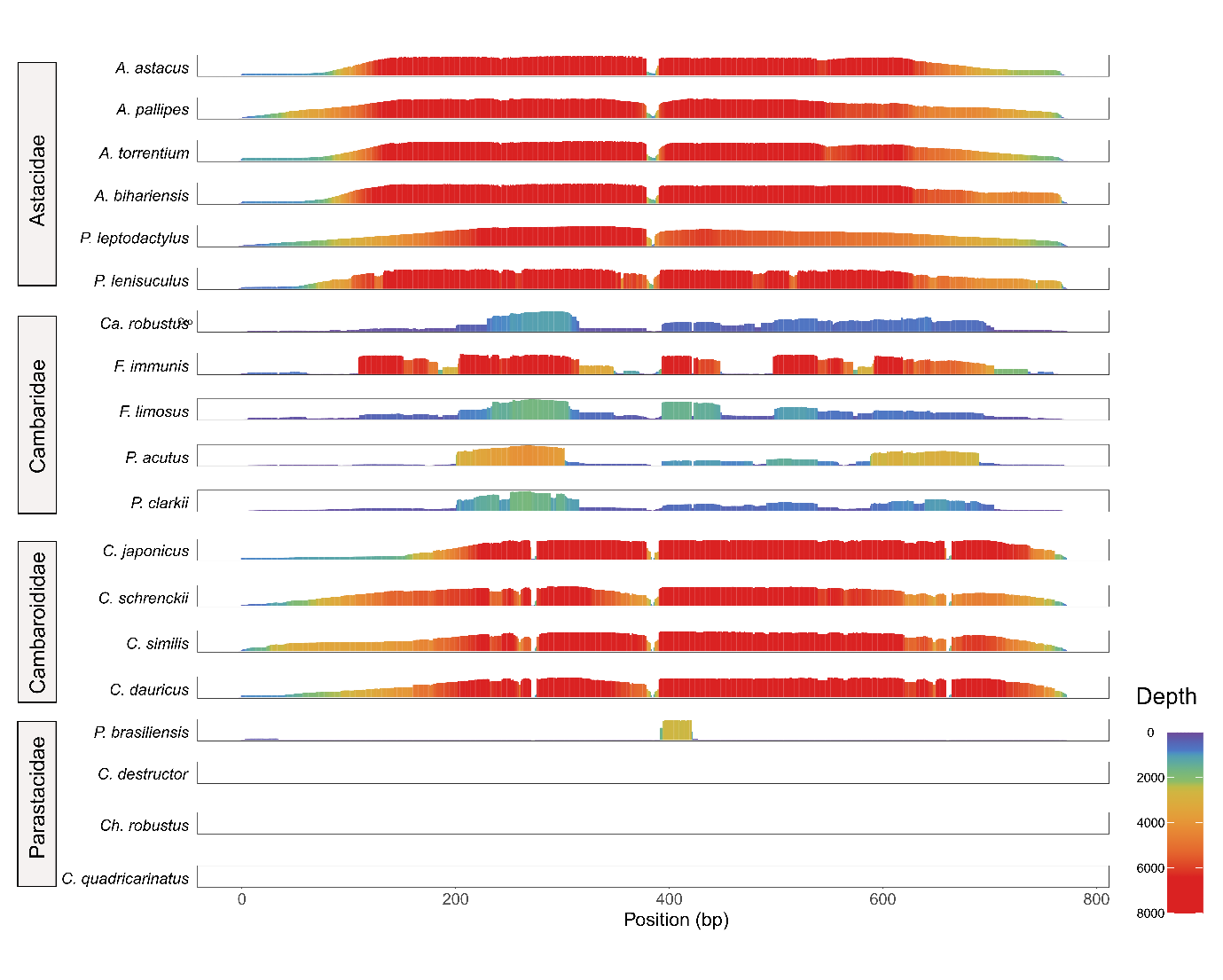


Supplementary figure 3. Colour enhanced profile of PlSAT3-411 satellite DNA family against each species. Different colours indicate coverage. The height of each bar indicates the coverage of base variant in the reads


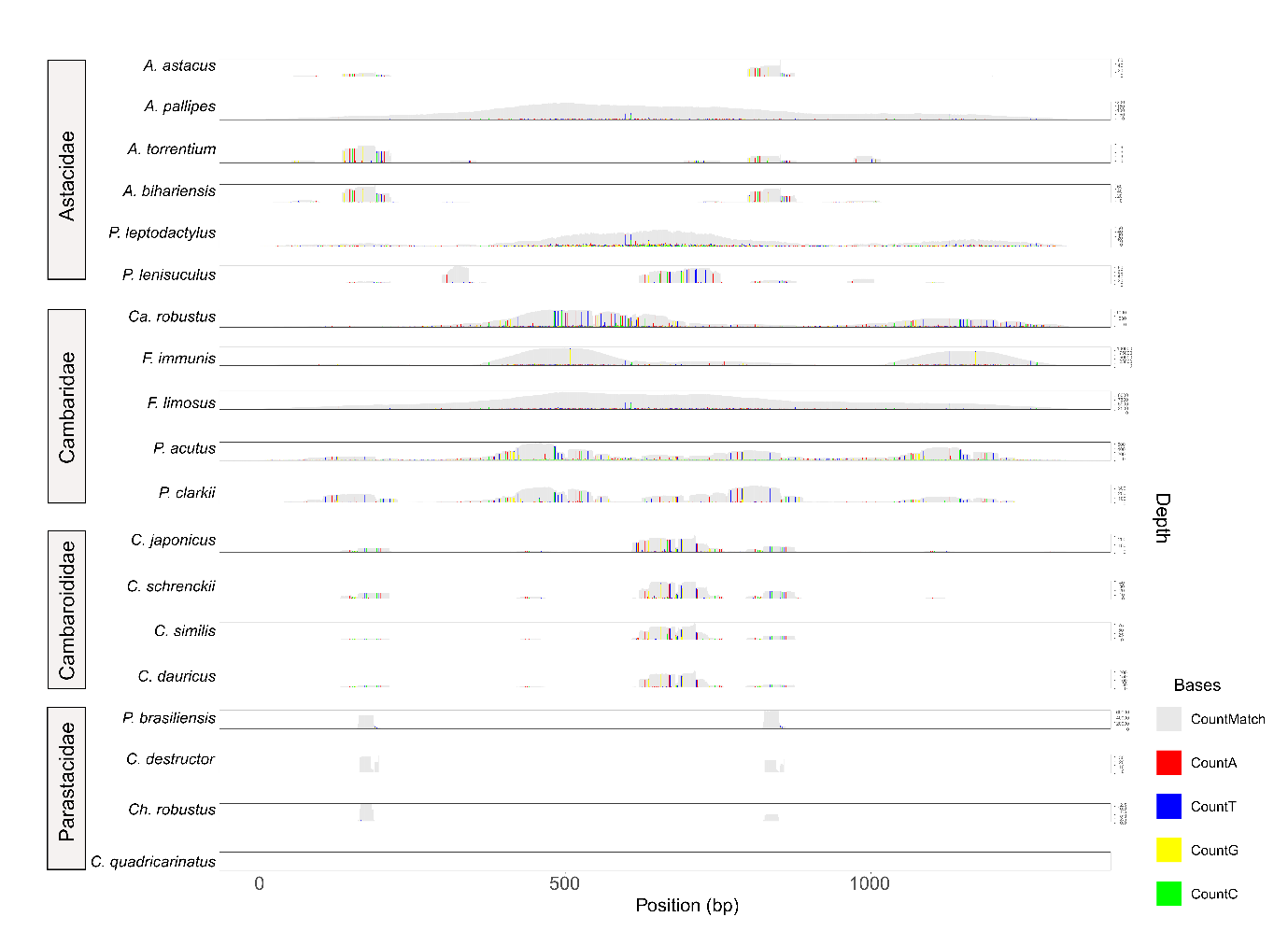


Supplementary figure 4. Variant repeat profiles of PlSAT57-664 satellite DNA family across the studied species. Different colours indicate A, T, C and G bases. The height of each bar indicates the coverage of each variant in the reads.


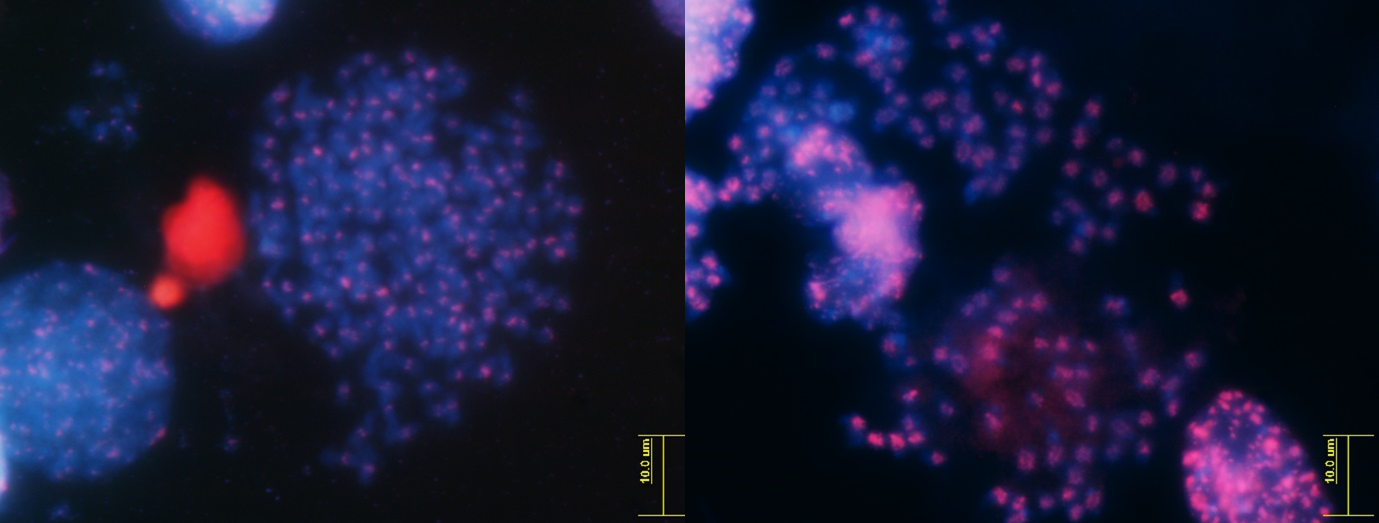


Supplementary figure 5. Localisation of PlSAT3-411 satellite repeat family (in red) on metaphase chromosomes of (A) A. torrentium and (B) P. leniusculus. Red signals represent the Cy3-labeled probe localisation, chromosomes are counterstained with DAPI. Scale bar = 10μm.

Supplementary table 1. Overview of the analysed species, including their genus, family, accession number of the reads, number of sequencing reads obtained in this study, mitochondrial genome accession number and genome size (Gb)

Supplementary table 2. Flow cytometry genome size measurement of haemolymph from Astacus astacus and Austropotamobius bihariensis obtained by PI fluorescence dye excitation with three chopping buffers.

| Species | Chopping buffer | Genome size (Mb) | Average genome size (Mb) |
| --- | --- | --- | --- |
| *A. astacus* | Galbraith et al. [31] | 17495 | 16891 |
| *A. astacus* | Galbraith et al. [31] | 19472 |  |
| *A. astacus* | Galbraith et al. [31] | 18894 |  |
| *A. astacus* | Otto et al. [32] | 15268 |  |
| *A. astacus* | Phospate buffer saline | 14944 |  |
| *A. astacus* | Phospate buffer saline | 15273 |  |
| *A. bihariensis* | Galbraith et al. [31] | 12240 | 11583 |
| *A. bihariensis* | Galbraith et al. [31] | 12170 |  |
| *A. bihariensis* | Phospate buffer saline | 10340 |  |

Supplementary table 3. Summary of clusters identified in individual RepeatExplorer runs for each crayfish species. For each cluster are indicated the unique cluster name (CL_unique), the supercluster classification, the cluster size, automatic annotation from RepeatExplorer, TAREAN annotation, the final manually curated annotation and genome proportion (%). For satDNA sequences the sequence, length (bp) and GC content (%) are indicated.

Supplementary table 4. Summary of clusters identified in the comparative RepeatExplorer run. For each cluster are indicated RepeatExplorer classification, TAREAN classification, total number of reads in a cluster and number of reads in a cluster belonging to a particular species, and correspondence to satDNA in individual clustering.

Supplementary table 5. Summary of Kruskal-Wallis tests comparing GC content and satDNA repeat length across genera and families. The table reports the tested variable, grouping factor, test statistic (Chi-squared), degrees of freedom (Df), and corresponding p-value for each comparison.

| Variable | Grouping | Chi-squared | Df | p-value |
| --- | --- | --- | --- | --- |
| Length | Genus | 340.9753 | 9 | 5.157e-68 |
| Length | Family | 325.8408 | 3 | 2.537e-70 |
| GC | Genus | 336.2363 | 9 | 5.252e-67 |
| GC | Family | 262.8178 | 3 | 1.105e-56 |

Supplementary table 6. Spearman rank correlation test between GC content and satDNA repeat length across freshwater crayfish families. The table reports the correlation value (Spearman’s rho) and corresponding p-value for each family.

|  | Family | Correlation | p-value |
| --- | --- | --- | --- |
| Satellites | Astacidae | -0.061 | 0.00397 |
| Satellites | Cambaridae | -0.098 | 0.00254 |
| Satellites | Cambaroididae | 0.022 | 0.49285 |
| Satellites | Parastacidae | -0.079 | 0.12717 |
| Minisatellites | Astacidae | -0.060 | 0.00542 |
| Minisatellites | Cambaridae | -0.005 | 0.89189 |
| Minisatellites | Cambaroididae | 0.030 | 0.35321 |
| Minisatellites | Parastacidae | -0.038 | 0.48598 |

Supplementary table 7. Results of pairwise Wilcoxon rank-sum tests comparing GC content and satDNA repeat length across genera and families. Adjusted p-values were calculated using the Bonferroni correction to control for multiple comparisons. Significance levels are indicated as follows: p < 0.05 *, p < 0.01 **, and p < 0.001 ***. The table includes the tested variable, grouping factor, compared groups, adjusted p-values, and significance annotations.

Supplementary file 1. Cluster_similarity.sh

Supplementary file 2. Cluster_similarity.R
